# Maternal metabolism affects endometrial expression of oxidative stress and *FOXL2* genes in cattle

**DOI:** 10.1371/journal.pone.0189942

**Published:** 2017-12-27

**Authors:** Audrey Lesage-Padilla, Niamh Forde, Mélanie Poirée, Gareth D. Healey, Corinne Giraud-Delville, Pierrette Reinaud, Caroline Eozenou, Anaïs Vitorino Carvalho, Laurent Galio, Mariam Raliou, Jean-François Oudin, Christophe Richard, I. Martin Sheldon, Gilles Charpigny, Pat Lonergan, Olivier Sandra

**Affiliations:** 1 UMR BDR, INRA, ENVA, Université Paris Saclay, Jouy en Josas, France; 2 School of Agriculture and Food Science, University College Dublin, Dublin, Ireland; 3 Institute of Life Science, Swansea University Medical School, Swansea University, Swansea, United Kingdom; University of Florida, UNITED STATES

## Abstract

Intensive selection for milk production has led to reduced reproductive efficiency in high-producing dairy cattle. The impact of intensive milk production on oocyte quality as well as early embryo development has been established but few analyses have addressed this question at the initiation of implantation, a critical milestone ensuring a successful pregnancy and normal post-natal development. Our study aimed to determine if contrasted maternal metabolism affects the previously described sensory properties of the endometrium to the conceptus in cattle. Following embryo transfer at Day 7 post-oestrus, endometrial caruncular (CAR) and intercaruncular (ICAR) areas were collected at Day 19 from primiparous postpartum Holstein-Friesian cows that were dried-off immediately after parturition (i.e., never milked; DRY) or milked twice daily (LACT). Gene quantification indicated no significant impact of lactation on endometrial expression of transcripts previously reported as conceptus-regulated (*PLET1*, *PTGS2*, *SOCS6*) and interferon-tau stimulated (*RSAD2*, *SOCS1*, *SOCS3*, *STAT1*) factors or known as female hormone-regulated genes (*FOXL2*, *SCARA5*, *PTGS2*). Compared with LACT cows, DRY cows exhibited mRNA levels with increased expression for *FOXL2* transcription factor and decreased expression for oxidative stress-related genes (*CAT*, *SOD1*, *SOD2*). *In vivo* and *in vitro* experiments highlighted that neither interferon-tau nor FOXL2 were involved in transcriptional regulation of *CAT*, *SOD1* and *SOD2*. In addition, our data showed that variations in maternal metabolism had a higher impact on gene expression in ICAR areas. Collectively, our findings prompt the need to fully understand the extent to which modifications in endometrial physiology drive the trajectory of conceptus development from implantation onwards when maternal metabolism is altered.

## Introduction

During recent decades, dairy cattle have been selected in order to improve milk production, leading to an associated decrease in fertility [1]. Greater milk production has been associated with physiological disturbances that accentuate negative energy balance during early lactation [2]. In high-producing dairy cows, fertilization rate has been shown to be as high as 80–90%, but only about 40% of inseminations result in the birth of a calf, with 70% of pregnancy failures occurring during the pre-implantation period [3]. Indeed, in a review of fertilisation and embryo quality in dairy cows, it was reported that only approximately 50% of embryos are still viable by Day 7 [4].Milk production has been shown to affect not only the production of oocytes but also embryo metabolism during conceptus free-floating life [5]. When the impact of lactation on oocyte quality is circumvented by the use of embryo transfer in lactating recipient females, pregnancy rate is improved [6–8]. Other experiments in which embryos were transferred at Day 7 post-oestrus have shown that pregnancy rate is reduced in lactating cows compared with heifers or dry cows [9–11]. In addition to decreasing fertility, alterations in maternal metabolism during pregnancy have also been reported to affect antenatal programming of production traits in offspring [12]. As a biological dynamic interface, the endometrium acts as a sensor of embryo quality that drives the developmental trajectory of the conceptus [13].Thus, endometrial quality appears critical for pregnancy as well as post-natal issues.

Implantation is a critical step of pregnancy [14] and is defined as the establishment of cellular and permanent interactions between a receptive endometrium and a competent and synchronised embryo [15]. At implantation, regulation of endometrial gene expression result from a combination of biological processes that involve actions of maternal signals, impact of embryo-secreted factors and cellular interactions between the conceptus and the endometrium. In ruminants including cattle, interferon-tau (IFNT) has been reported as the major signal of maternal recognition of pregnancy [16,17]. The spatio-temporal secretion of IFNT is restricted to the trophectoderm during the elongation phase of the conceptus [18]. In the ovine and bovine species, expression patterns of endometrial genes have been intensively investigated by our laboratory and others using in vitro and in vivo experimental models associated with high-throughput analyses and candidate gene approaches. Determining variations in gene expression during estrous cycle and early pregnancy allow a classification of endometrial genes in three major categories namely (i) conceptus regulated gene expression that involves IFNT, making these genes members of classical and non-classical interferon-regulated genes such as the STAT1-SOCS pathway thought to play critical functions during implantation in cattle [19,20], (ii) conceptus regulated and IFNT-independent expression that is modified by still unknown or already identified conceptus-produced factors (e. g. cortisol, prostaglandins [21]) such as *SOCS6* [22] and *PLET1* [23], two genes highly expressed in the luminal epithelial cells when apposition initiates, and (iii) expression that is not affected by the presence of the conceptus but is variable across the estrous cycle (e. g. under the influence of female hormones including ovarian steroids [16,24,25] such as the scavenger receptor *SCARA5* [23], the key enzyme in prostaglandin biosynthesis *PTGS2* [26,27], and *FOXL2*, a key transcription factor of the ovary and recently characterized in the bovine endometrium [28]. These three categories of regulated genes contribute to endometrial receptivity, conceptus elongation and implantation and their perturbed expression may lead to detrimental effects on establishment, progression and outcome of pregnancy [13].

After calving, dairy cows undergo metabolic adjustments that are associated with the transition from pregnancy to lactation. In order to meet the increasing demand for energy in early lactation, shifts in nutrient distribution have been described [29]. In this context, the imbalance between reactive oxygen species (ROS) production and the availability of antioxidant defences during early lactation may expose cows to oxidative stress observed in blood plasma [30]. In mice, ROS may negatively impact embryonic metabolism leading to pregnancy complications and long-term modifications of offspring metabolism [31]. Activity of antioxidant enzymes involved in the regulation of oxidative stress (catalase, CAT; super oxide dismutase 1, SOD1; super oxide dismutase 2, SOD2) has been reported to vary in ovine endometrium during the peri-implantation period [32] and with endocrine milieu in the endometrium of beef cows at Day 7 of the oestrous cycle [33]. Interestingly, in immortalized granulosa cells, *SOD2* gene expression has been shown to be affected by FOXL2 [34]. Nevertheless, when variations in maternal metabolism are considered, data relating to the regulation of these enzymes in the endometrium are lacking.

Considering this background, our study aimed to determine if maternal metabolism has distinct effects on the two areas that constitute the bovine endometrium at the time of implantation. In ruminants, endometrial morphology is characterised by caruncular (CAR) and intercaruncular areas (ICAR), both essential for supporting pregnancy. Caruncular endometrium is represented by small sparse aglandular structures, whereas intercaruncular endometrium contains endometrial glands the secretions of which are critical for conceptus elongation [35]. High-throughput data have highlighted that endometrial CAR and ICAR areas exhibit a site-specific response to the presence of the implanting conceptus in normal or perturbed pregnancies [22,23,28,36–38]. In this study, we used a novel model of age-matched pregnant primiparous postpartum lactating or non-lactating dairy cows combined with embryo transfer [39]. At Day 19 post-oestrus, we analysed the expression of genes known to be deeply affected in the endometrium when implantation occurs (i) conceptus-regulated and IFNT-Stimulated genes (ISG) namely *RSAD2* [23,40] and three members of the STAT-SOCS transduction pathway (*STAT1*, *SOCS1*, *SOCS3)* [22,41]; (ii) conceptus-regulated but IFNT-independent genes, namely *PLET1*, *PTGS2* and *SOCS6*, and (iii) female hormone-regulated genes including *SCARA5* and *FOXL2* as well as *CAT*, *SOD1* and *SOD2*, three genes encoding enzymes with critical functions in oxidative stress regulation. For these three genes, expression in the bovine endometrium during implantation process and regulation by IFNT was first documented.

## Materials and methods

### Animal models

All experiments were conducted in accordance with the European Community Directive 2010/63/EU revising Directive 86/609/EEC on the protection of animals used for scientific purposes. Animal procedures described in Experiments 1 and 2 were licensed by the Department of Health and Children, Ireland, in accordance with the Cruelty to Animals Act (Ireland 1876) and were sanctioned by the Animal Research Ethics Committee of University College Dublin. Animals in experiments 1, 2 and 4 were slaughtered and processed as part of the normal work of a commercial abattoir. Animal procedures reported for endometrial biopsy collection were approved by the Ethical Committee of Animal Experimentation of INRA and AgroParisTech (CEEA 45 “COMETHEA”; reference APAFIS#596) with authorization subsequently granted by the French Ministry of Education and Scientific Research (reference 2015050417061568).

### Experiment 1: Impact of contrasted maternal metabolism on endometrial gene expression modifications at implantation

Twenty-one primiparous Holstein-Friesian cows and six Holstein-Friesian heifers (HEIF) were enrolled onto this study as previously described [39]. At calving, cows were randomly assigned to one of two groups: lactating (LACT, n = 11) or non-lactating (DRY, n = 10). From calving, animals in the LACT group were milked twice per day (0700 and 1600 hours), while those in the DRY group were dried-off immediately after calving (i.e., never milked) as described by Forde *et al*. [39]. All animals were synchronised 65 to 75 days after calving using an EAZI-BREED^™^ CIDR^®^ cattle insert in conjunction with prostaglandin F2α (Zoetis, Parsippany-Troy Hills, New Jersey, USA). Single grade 1 Day 7 embryos from superovulated donor heifers were transferred to LACT and DRY cows. As a control, HEIF were artificially inseminated. Blood samples were taken from the jugular vein into plain red-topped vacutainer tubes for serum collection. Progesterone serum concentrations on Day 19 were determined according to Carter *et al*. [42].

The day of oestrus was considered Day 0 and all females were slaughtered at Day 19, coinciding with the initiation of implantation in cattle. Uteri were collected and flushed and, when present, recovered concepti were observed by microscopy to confirm the stage of development [43]. From pregnant animals (HEIF n = 4; LACT n = 5; DRY n = 8), endometrial CAR and ICAR areas were dissected from the uterine horns ipsilateral to the corpus luteum as previously described [23]. For each type of endometrial area, a pool of tissue representative of the whole horn was used for protein or total RNA extraction. Tissue samples were snap frozen in liquid nitrogen and stored at -80°C prior to extraction.

### Experiment 2: Expression of oxidative stress enzymes during the oestrous cycle and early pregnancy

In order to provide insights on gene expression of antioxidant enzymes in bovine endometrium, cross-bred heifers were collected at two days of the oestrous cycle (active luteal phase and luteolysis) as well as during pregnancy recognition and implantation as previously published [28]. Heifers were synchronized and artificially inseminated as formerly described [44]. The day of oestrus was considered Day 0 and heifers were slaughtered on Day 16 (cyclic: n = 5; pregnant: n = 4) or on Day 20 (cyclic: n = 6; pregnant: n = 5). Uteri were collected from pregnant and cyclic animals, they were flushed and, when present, recovered concepti were observed by microscopy to confirm the stage of development [43]. Endometrial CAR and ICAR areas were dissected from the uterine horns ipsilateral to the corpus luteum [23]. Tissue samples were snap frozen in liquid nitrogen and stored at -80°C prior to extraction.

### Experiment 3: FOXL2 overexpression in primary cultures of bovine endometrial cells

In order to determine if FOXL2 regulates gene expression of enzymes involved in the regulation of oxidative stress, we collected endometrial biopsies during the active luteal phase, at Day 15 of the oestrous cycle when endometrial expression of *FOXL2* was reported to be low [28]. Three Holstein heifers (n = 3) were sampled, that had been synchronized using Creastar method, EAZI-BREED^™^ CIDR^®^ cattle insert (Zoetis). From each heifer, one endometrial biopsy was sampled and used for deriving primary cultures of fibroblasts and glandular epithelial cells as previously reported [23]. For each heifer, fibroblasts and epithelial cells were cultured separately, leading to 3 independent biological replicates for each cell type. Endometrial cells were cultured for 24 h on 12-well plates (TPP, Trasadingen, Switzerland). Fibroblasts and epithelial cells were transfected with 0.5 μg of pSG5 plasmid (mock) or pSG5-FOXL2 plasmid (kindly provided by M. Pannetier; INRA, Jouy-en-Josas, France; [45]), using CombiMag transient reagent (Oz Biosciences, Marseille, France) according to the manufacturer's instructions. Transfected cells were lysed after 24 h using Trizol reagent (Life Technologies, Carlsbad, California, USA). Cell lysates were frozen and stored at -80°C for gene expression analyses.

For determining FOXL2 localization by immunocytochemistry, fibroblasts and glandular epithelial cells were cultured using a Lab-tech chamber slide system (Sigma-Aldrich, St. Louis, Missouri, USA) and transiently transfected as described above. Cells were fixed for 10 min at room temperature in PAF 4% (Sigma-Aldrich) and washed in PBS (Sigma-Aldrich). Slides were incubated in citrate buffer (sodium citrate 0.01 M, pH 6) for 5 min at room temperature then for 10 min at 80°C. Endogenous peroxidase activity was quenched by 0.01% H_2_O_2_ (Sigma-Aldrich) treatment for 30 min. Cells were incubated with a rabbit anti-FOXL2 purified antibody (dilution 1:250; [28]) in phosphate buffer (0.1 M, pH 7.4 with 2% bovine serum albumin (BSA) and 1% Normal Donkey Serum) overnight at 4°C. After washes in phosphate buffer (0.1 M, pH 7.4 with 2% BSA), slides were incubated with an anti-rabbit biotinylated secondary antibody (dilution 1:1000; Ab6720, Abcam, Cambridge, UK) for 1 h at room temperature. Cells were washed in phosphate buffer containing 2% BSA and incubated for 1 h with ABC Vector kit (Vectastain Elite ABC kit, Vector Labs, Peterborough, UK) in Tris buffer (Tris 50 mM, NaCl 0.15 M, pH 7.5). Slides were washed, incubated for 5 min with Diaminobenzidine-Nickel in Tris buffer then they were mounted with Eukitt medium (Sigma-Aldrich). Nanozoomer Digital Pathology System was used to obtain images that were analysed using the NDP View software (Nanozoomer Digital Pathology Virtual Slide software, Hamamatsu, Japan).

### Experiment 4: Treatment of bovine endometrial cell cultures with interferon-tau (IFNT)

In order to evaluate the impact of IFNT on the expression of genes encoding antioxidant enzymes, we used primary populations of epithelial and fibroblast cells that were isolated from bovine endometrium collected from cyclic mixed breed beef cows on Day 11–17 post-oestrus, as previously described [46]. During this period of the estrous cycle, endometrium has been shown to strongly react to conceptus secretions including IFNT [20,44]. The endometrial cells were cultured in medium containing RPMI-1640 Medium (Sigma-Aldrich, Saint-Quentin Fallavier, France), supplemented with 10% heat inactivated Fetal Bovine Serum (Sigma-Aldrich), 1% Penicillin-Streptomycin (Sigma-Aldrich), 1% Amphotericin B (Sigma-Aldrich). The epithelial and fibroblast cells were each treated with control medium or medium containing ovine recombinant IFNT (100 ng/ml or 1000 ng/ml) for 30 min, 2 h or 24 h [23,41]. Bovine endometrial cells were washed with Dulbecco’s Phosphate Buffered Saline with MgCl_2_ and CaCl_2_ (Sigma-Aldrich) and lysed using Trizol reagent (Life Technologies). Cell lysates were frozen and stored at -80°C before analysis. Data were generated using stromal and epithelial cells isolated from four independent animals.

### Protein extraction and western blot analyses

FOXL2 protein level was investigated from endometrial ICAR and CAR protein extracts described in experiment 1 (n = 3 animals/condition). Frozen ground tissue (250 mg) was dispersed and sonicated in 1 ml of a cold lysis buffer at pH 7.4 containing 50 mM Hepes, 150 mM NaCl, 5 mM EDTA, 16 mM 3-((3-cholamidopropyl) dimethylammonio)-2-hydroxy-1-propanesulfonate, 1mM benzamidine-HCl, 1mM phenylmethylsulfonyl fluoride, 10 μg/ml soybean trypsin inhibitor, 10 μg/ml leupeptin and 10 μg/ml aprotinin. Protein quantification was performed with the Bio-rad protein assay (Bio-Rad, Hercules, California, USA) using BSA as the standard (Sigma Aldrich).

Western blot immunoassays were processed with 25 μg of total protein extract electrophoresed in SDS–10% polyacrylamide gel and onto a Hybond-P, polyvinylidene difluoride membrane (Trans-blot® Turbo Midi Size PVDF Membrane; Bio-Rad) with Trans-blot® Turbo (Bio-Rad) system. Membranes were first incubated with a rabbit anti-FOXL2 purified antibody generated against a peptide (sequence: WDHDSKTGALHSRLDL) corresponding to the C-terminal conserved region of mammalian FOXL2 (CASLO Laboratory, Lyngby, Denmark; [28]) in a solution containing 20 mM Tris-HCl, 500 mM NaCl, (pH 7.6), 0.1% (wt/vol) Tween 20 containing 5% (wt/vol) low-fat milk (2 μg antibody/ml). Then, membranes were incubated with a biotinylated donkey anti-rabbit IgG antibody (Jackson ImmunoResearch, West Grove, Pennsylvania, USA). Blots were developed for 1 h using the Vectastain Elite ABC peroxidase complex 1:1000 diluted (Vectastain Elite ABC kit; Vector Labs). Immunoreactive signals were revealed with ECL2 western blotting detection kit (Thermo Fisher Scientific, Waltham, Massachusetts, USA). After stripping, GAPDH protein was assessed as a loading control using a rabbit polyclonal anti-GAPDH antibody (Sigma-Aldrich) and donkey peroxidase-conjugated anti-rabbit antibody (Interchim, San Diego, California, USA). The molecular weights of the identified proteins were calculated using the ProSieve HyperPage Meridian (Bioline, London, UK).

### Total RNA extraction

Total RNA were isolated from biological samples described in experiments 1, 2, 3 and 4 by homogenization using Trizol reagent (Life Technologies) as published previously [23]. All RNA samples were purified using Qiagen columns integrating a DNAse step (RNeasy mini kit; Qiagen, Hilden, Germany). Quality and integrity of purified total RNA were determined using an Agilent 2100 Bioanalyzer (@BRIDGe-ICE platform, INRA, Jouy-en-Josas, France). Total RNA samples were stored at -80°C prior to analysis.

### Real time RT-PCR

Purified total RNA samples were used for gene quantification using Real Time-PCR (RT-qPCR). One μg of total RNA was reverse transcribed into cDNA with Superscript II enzyme (Life Technologie) in a 20 μl-volume. RT-qPCR reactions were carried out with Master Mix SYBR Green and Step One Plus system (Applied Biosystem, Foster city, California, USA). Primers (Eurogentec, Liège, Belgium) were designed (Primer express version 2.0 software; Applied Biosystem) to specifically amplify housekeeping genes (*C2ORF29*, *GAPDH*, *RPL19*, *SLC30A6*, *SUZ12*) and target genes (S1 Table).

### Statistical analyses

Quantification of the amount of target mRNA relative to that of normalizer genes was calculated using Qbase plus software (Biogazelle; Zwijnaarde, Belgium) according to the relative standard curve method [47]. Normalized PCR data were processed using the Kruskal-Wallis method using the plug-in Coin (COnditional INference procedures in a Permutation Test Framework; Package Rcmdr) associated with pairewise t-test (R application V3.0.1). Principal Component Analysis (PCA) were carried out in Excel using the XLSTAT software (Addinsoft, Paris France).

## Results

### Endometrial expression of conceptus-regulated genes is not affected by maternal metabolism at implantation

**Fig 1:**
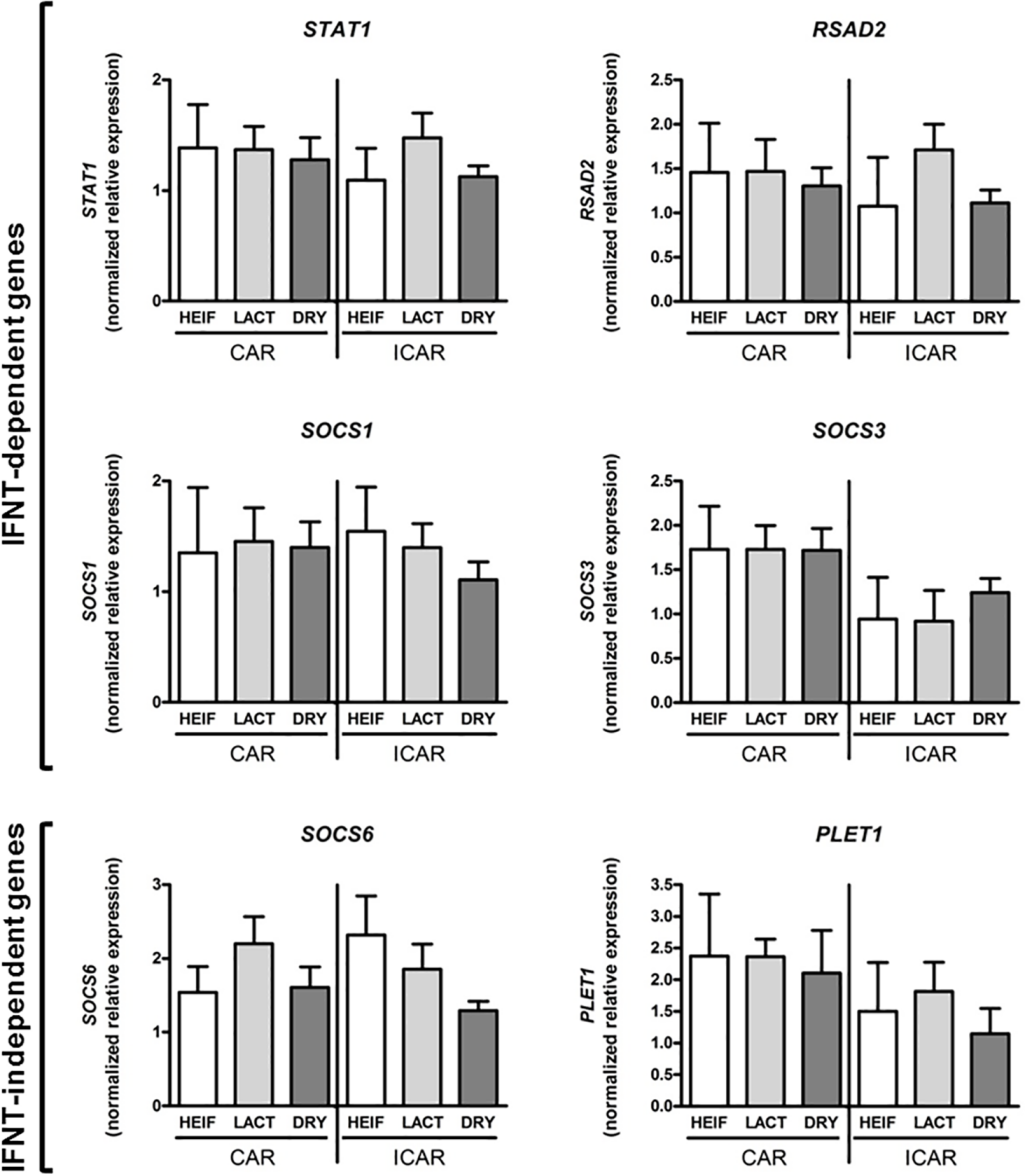
Expression of conceptus-regulated genes in bovine endometrium at implantation. Caruncular (CAR) and intercaruncular (ICAR) endometrium was collected from Holstein heifers (HEIF, white; n = 3), lactating (LACT, light grey; n = 5) and non-lactating (DRY, dark grey; n = 8) cows at Day 19 post-oestrus. Gene expression was quantified by real-time RT-PCR using *C2ORF29*, *GAPDH* and *SLC30A6* as housekeeping genes determined by Qbase plus software. Data are means ± SD.

During early pregnancy, the conceptus impacts endometrial physiology through cellular contact and secretions that include IFNT in ruminants. We investigated the impact of contrasted maternal metabolism on transcriptional expression of a selection of conceptus-regulated genes in endometrial CAR and ICAR areas. Amongst conceptus-regulated genes, *RSAD2*, *STAT1*, *SOCS1* and *SOCS3* are IFNT-dependent genes [22,41,48] whereas *SOCS6* and *PLET1* are IFNT-independent genes [22,23]. Variation in maternal metabolism did not significantly affect endometrial expression of these genes on Day 19 of pregnancy (Fig 1).

### *FOXL2* gene expression at implantation varies with maternal metabolism

**Fig 2:**
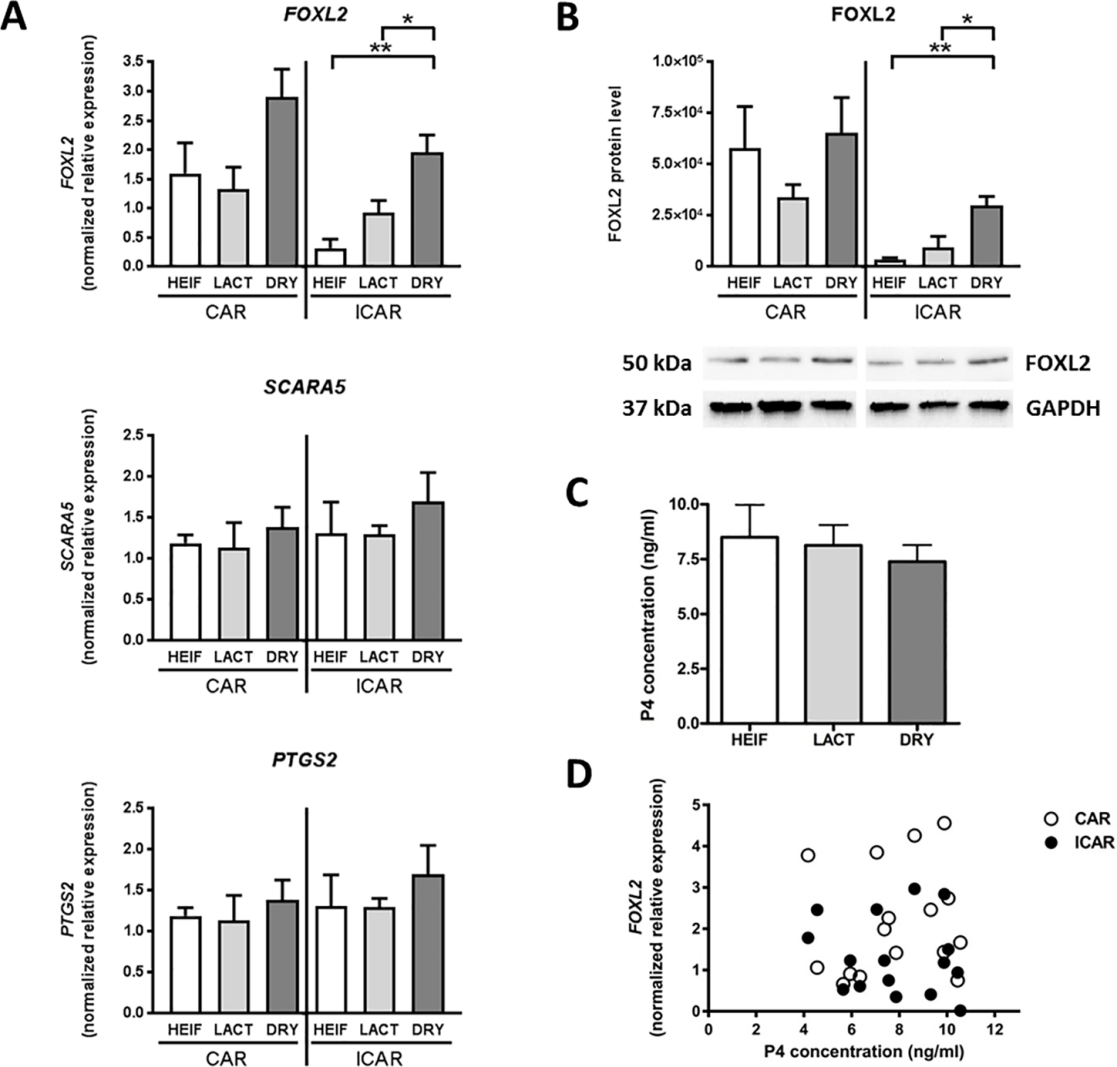
Expression of maternally-regulated genes in bovine endometrium at implantation. Caruncular (CAR) and intercaruncular (ICAR) endometrium and blood samples for progesterone analysis were collected from Holstein heifers (HEIF, white; n = 3), lactating (LACT, light grey; n = 5) and non-lactating (DRY, dark grey; n = 8) cows on Day 19 post-oestrus. (A) *SCARA5*, *PTGS2* and *FOXL2* mRNA expression in bovine endometrium quantified by real-time RT-PCR using *C2ORF29*, *GAPDH* and *SLC30A6* as housekeeping genes determined by Qbase plus software. (B) Quantification of FOXL2 protein by western blotting normalized to GAPDH protein level. (C) Circulating progesterone concentration in Holstein heifers, lactating and non-lactating cows. (D) Correlation between *FOXL2* mRNA expression and circulating progesterone concentration in CAR (white circle; n = 16) and ICAR (black circle; n = 16) areas. Data are means ± SD (P<0.01 **; P<0.05 *).

Maternal regulation of endometrial gene expression has been shown to be essential for endometrial receptivity and subsequent embryo implantation. In this model of contrasted metabolism, we investigated expression of three endometrial genes known to be regulated by maternal signals but not by the conceptus, namely *SCARA5* [23], *PTGS2* [26,27] and *FOXL2* [28]. Maternal metabolism had no significant effect on *SCARA5* and *PTGS2* mRNA levels in bovine endometrium. *FOXL2* transcript expression was significantly higher in ICAR areas of DRY cows compared with those of LACT cows (2.1 fold, P < 0.05) and HEIF (6.1 fold, P < 0.01) groups. No significant effect of the maternal metabolism on *FOXL2* expression was noticeable in CAR areas (Fig 2A). Similarly, at the protein level, regulation of FOXL2 expression was seen with a significantly higher expression of FOXL2 in ICAR areas of the DRY group compared with the two other groups (DRY *vs* LACT: 3.2 fold, P < 0.05; DRY *vs* HEIF: 8.1 fold, P < 0.01; Fig 2B). There was no difference in plasma P4 concentrations between groups and *FOXL2* mRNA expression in CAR and ICAR areas was not correlated with P4 blood concentrations (Fig 2C and 2D).

### Endometrial *CAT*, *SOD1* and *SOD2* expression varies with maternal metabolism at implantation

**Fig 3:**
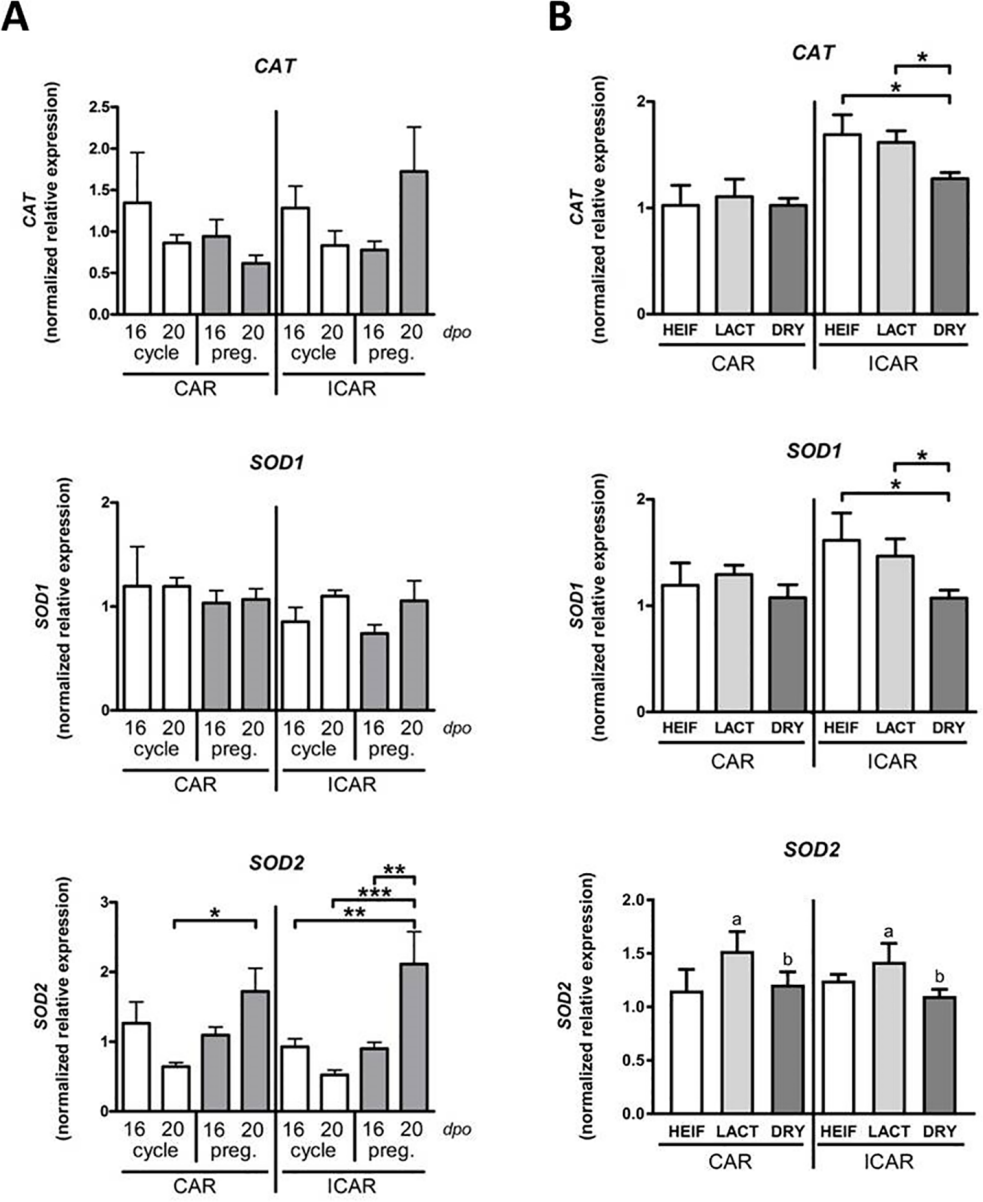
Expression of *CAT*, *SOD1* and *SOD2* genes in bovine endometrium. (A) *CAT*, *SOD1* and *SOD2* mRNA expression in cyclic and pregnant endometrium on Days 16 and 20 post oestrous. Caruncular (CAR) and intercarncular (ICAR) endometrium was collected from cyclic (white; n = 5 at Day 16 and n = 6 at Day 20) and pregnant (grey; n = 4 at Day 16 and n = 5 at Day 20) cross-bred beef heifers. Gene expression was quantified by real-time RT-PCR using *SUZ12* and *SLC30A6* as housekeeping genes determined by Qbase plus software. (B) *CAT*, *SOD1* and *SOD2* mRNA expression in bovine endometrium on Day 19 post-oestrus. CAR and ICAR areas were collected from Holstein heifers (HEIF, white; n = 3), lactating (LACT, light grey; n = 5) and dried-off (DRY, dark grey; n = 8) cows at Day 19 post-oestrus. Gene expression was quantified by real-time RT-PCR using *C2ORF29*, *GAPDH* and *SLC30A6* as housekeeping genes determined by Qbase plus software. Data are means ± SD (P<0.001 ***; P < 0.01 **; P<0.05 *).

We first determined if *CAT*, *SOD1* and *SOD2* transcripts were expressed in bovine endometrium and regulated during oestrous cycle and early pregnancy (Fig 3A). In CAR and ICAR areas, endometrial expression of *CAT* and *SOD1* mRNA did not significantly differ between Day 16 and Day 20 in cyclic or in pregnant heifers. At Day 20 of pregnancy, endometrial expression of *SOD2* mRNA was significantly higher when compared with Day 20 of the oestrous cycle (4.0 fold, P < 0.001 in ICAR; 2.7 fold, P < 0.05 in CAR) or with Day 16 of pregnancy (2.3 fold, P < 0.01 in ICAR).

In our model of contrasted maternal metabolism (Fig 3B), *CAT* and *SOD1* mRNA expression was significantly lower in endometrial ICAR areas of DRY cows compared with those of LACT cows or heifers (0.78 and 0.73 fold respectively; P < 0.05). Similarly *SOD2* mRNA expression was reduced in the DRY group compared with the LACT group when endometrial CAR and ICAR areas were considered simultaneously (0.78 fold, P < 0.05).

### *CAT*, *SOD1* and *SOD2* mRNA levels are regulated neither by interferon-tau nor by FOXL2

**Fig 4:**
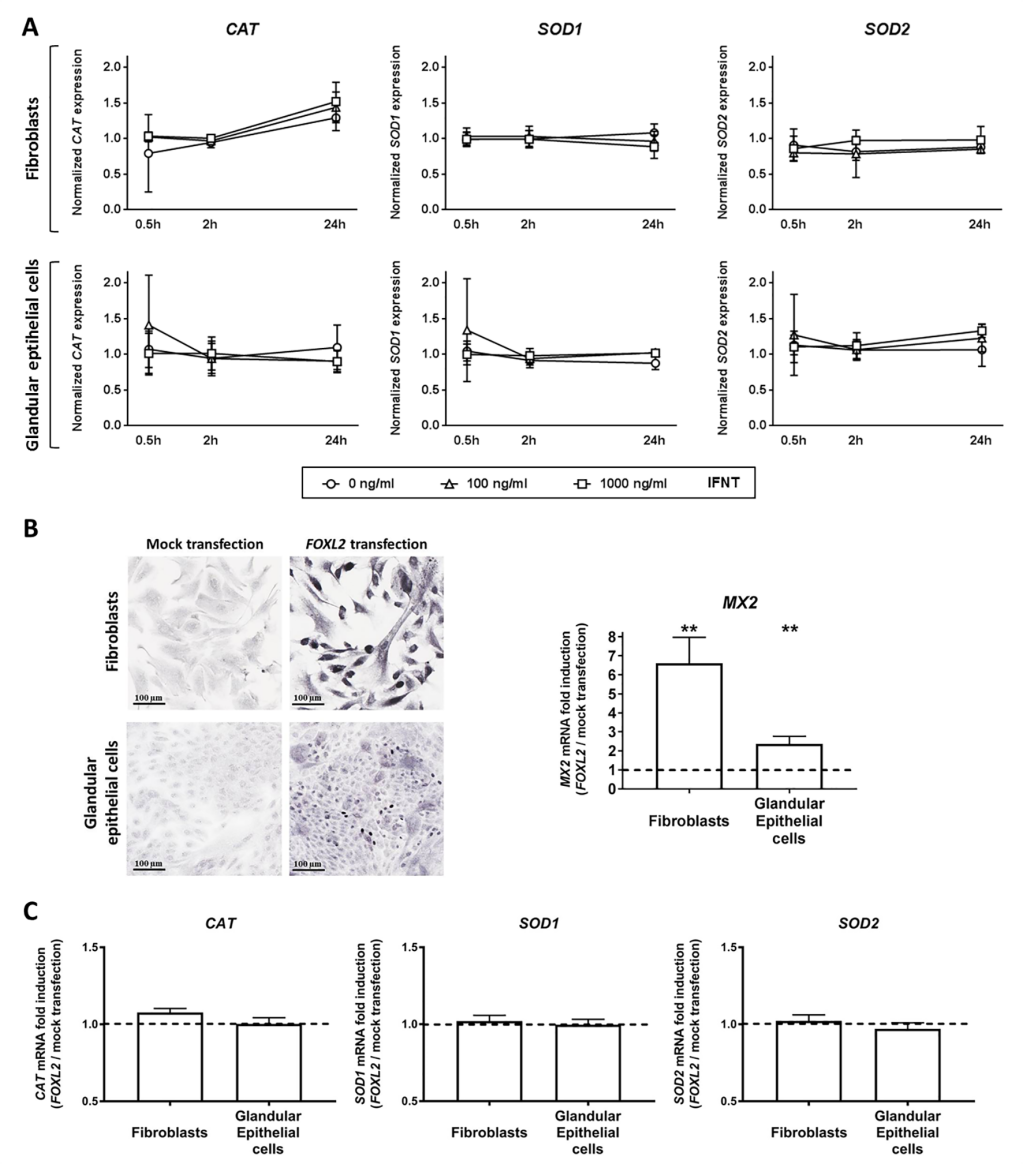
Regulation of *CAT*, *SOD1* and *SOD2* gene expression by interferon-tau (IFNT) and FOXL2 in bovine endometrial cells. (A) Regulation of *CAT*, *SOD1* and *SOD2* mRNA expression by IFNT in primary cultures of bovine endometrial cells. Fibroblasts or glandular epithelial cells were incubated with 0 (circle), 100 (triangle) or 1000 ng/ml (square) of IFNT for 30 min, 2 h and 24 h. (B) Left panel. FOXL2 sub-cellular localization in fibroblasts and glandular epithelial cells in mock-transfection and *FOXL2*-transfection. Scale bar: 100 μm. Right panel. *MX2* mRNA expression in primary cultures of bovine endometrial cells overexpressing *FOXL2* by transient transfection. (C) *CAT*, *SOD1* and *SOD2* mRNA expression in primary cultures of bovine endometrial cells overexpressing *FOXL2* by transient transfection. Transcript expression is presented as a ratio between “*FOXL2* transfection” condition and “mock transfection” condition (dashed line). Gene expression was quantified by real time RT-PCR using *C2ORF29*, *RPL19* and *SLC30A6* as housekeeping genes determined by Qbase plus software. Data are means ± SD.

To determine if *CAT*, *SOD1* and *SOD2* gene expression was regulated by IFNT, primary cultures of bovine endometrial cells (fibroblasts and glandular epithelial cells) were treated in the presence of 0, 100 or 1000 μg/ml of IFNT for 30 min, 2 h or 24 h. In our previous report, these cultures were shown to be IFNT-responsive [41] but no significant effect of IFNT was detected on mRNA expression of antioxidant enzymes (Fig 4A).

Since FOXL2 was reported to regulate oxidative stress in ovary-related cells [34,49], the effect of FOXL2 on *CAT*, *SOD1* and *SOD2* gene expression was investigated. Overexpression of FOXL2 was reached by transiently transfecting primary cultures of bovine endometrial cells (fibroblasts and glandular epithelial cells). FOXL2 nuclear localization was confirmed by immunocytochemistry in primary cultures of transfected fibroblasts and glandular cells (Fig 4B, left panel). Biological effect of FOXL2 overexpression was confirmed by quantifying *MX2* gene expression. Significant up-regulation of *MX2* mRNA levels was detected in fibroblasts (6.61 fold; P < 0.01) and in glandular epithelial cells (2.36 fold, P < 0.01) (Fig 4B, right panel). FOXL2 overexpression did not significantly affect *CAT*, *SOD1* or *SOD2* mRNA levels in either cell type (Fig 4C).

### Principal component analysis (PCA) of endometrial gene expression in LACT, DRY and HEIF groups

**Fig 5:**
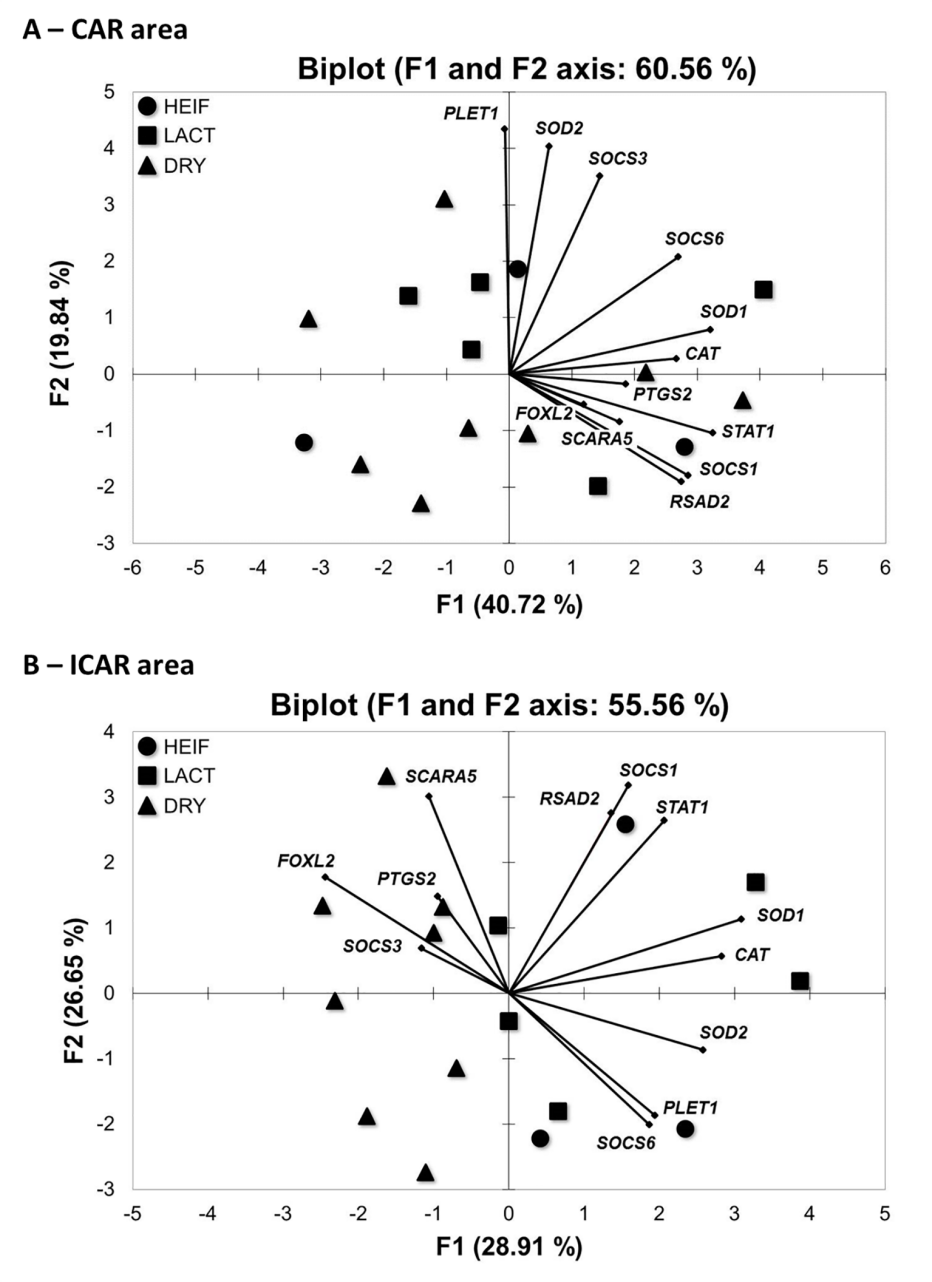
Gene clustering in the bovine endometrium. Principal component analysis (PCA) was run to distinguish between Holstein heifers (circle; n = 3), lactating cows (square; n = 5) and non-lactating (triangle; n = 8) cows at Day 19 post-oestrus based on mRNA levels of genes expressed in CAR (A) and ICAR (B) areas. Gene expression was quantified by real-time RT-PCR using *C2ORF29*, *GAPDH* and *SLC30A6* as housekeeping genes determined by Qbase plus software.

A two-dimensional PCA was carried out when considering gene expression of the various genes that were analysed according to maternal metabolism. PCA highlighted distinct gene distribution for each endometrial area when maternal metabolic status varies (Fig 5A and 5B). Our PCA analysis did not allow the discrimination of LACT, DRY and HEIF groups in CAR areas (Fig 5A). In contrast, the F1 axis discriminates DRY cows from LACT cows and from heifers in ICAR areas (Fig 5B).

## Discussion

In dairy cattle, poor reproductive performance results in greater economics losses for the farmer. Approximately two-thirds of pregnancy failures are believed to occur during the pre-implantation period [3]. As the maternal tissue layer dedicated to the molecular and cellular interactions with the implanting conceptus, the uterine endometrium drives development of the embryonic disk and elongation of the extra embryonic tissues [13,50]. Environmental stress or maternal physiology disturbances have been reported to affect endometrial function that may in turn impact conceptus development during the peri-implantation period with long-term consequences. In high-producing dairy cows, lactation has been shown to generate metabolic stress due to energy demands associated with milk production [39,51,52]. The present study aimed to determine if modifications in maternal metabolism observed between lactating and non-lactating primiparous cows [39,52,53] impact the expression of genes involved in the regulation of endometrial physiology at the initiation of implantation.

Mammalian endometrium has been demonstrated to act as a sensor that can adapt its physiology to variations in embryo quality [54,55]. Recent data in humans have suggested that maternal alterations in endometrial biosensor properties skew the biological response of this tissue to embryo signals [54]. We investigated the impact of a contrasted maternal metabolism on endometrial mRNA levels of conceptus-regulated genes we formerly classified as IFNT-stimulated genes (*RSAD2*, *SOCS1*, *SOCS3* and *STAT1*,) or IFNT-independent genes (*PLET1* and *SOCS6*) in cattle [23,37,41]. We analysed the caruncular (CAR; gland-free zones where placentation occurs) and intercaruncular (ICAR; containing endometrial glands that secrete histotroph critical for extra-embryonic tissue elongation; [35]) areas of the bovine endometrium that have been shown to respond differentially to the presence of the conceptus [22,23,36–38,41]. Our data showed that transcriptional expression of this set of conceptus-regulated genes was not significantly affected in either endometrial area when lactating cows were compared with non-lactating cows. Absence of significant *RSAD2*, *STAT1* and *SOCS1* transcript regulation in endometrium is consistent with formerly published analyses that were run with a dairy cattle model of contrasted metabolism with timed AI [53]. Our data support the notion that lactation-induced changes in metabolic status do not seem to significantly compromise the sensor property of endometrium and its ability to response to embryonic signals when implantation occurs.

Based on data generated in sheep, tight regulation of cellular reactive oxygen species (ROS) at the conceptus-endometrium interface appears critical for implantation and pregnancy outcome. SOD1, SOD2 and CAT enzymes have been suggested to control O_2_− and H_2_O_2_ generation as a means of limiting detrimental impact of oxidative and harmful ROS [32]. The present study showed that *CAT*, *SOD1* and *SOD2* transcripts are expressed in endometrial CAR and ICAR areas of cyclic and pregnant heifers and *SOD2* mRNA levels were significantly up-regulated in ICAR areas of bovine endometrium at Day 20 of pregnancy. Our data are consistent with previous data reporting an increased amount of SOD2 in ovine intercaruncular endometrium at implantation [56]. Consequently, association between conceptus implantation and increased *SOD2* gene expression or activity in the endometrium appears to be a conserved feature that could be necessary for the correct progression of pregnancy in ruminants. Interestingly, we showed that increase in *SOD2* gene expression is not a consequence of IFNT action, demonstrating that neither *SOD2* nor *SOD1* or *CAT* can be classified as interferon-stimulated genes.

Oxidative stress results from the balance between the production of ROS and the capacity of antioxidant mechanisms to neutralize them in tissues and in blood. Continuous production of ROS results from normal metabolic processes, but it is known that this production rate varies with alterations of metabolic demand. In clinically healthy dairy cows, antioxidant status in the plasma compartment has been shown to reflect variations in maternal metabolism, during the periparturient and lactation periods [57,58]. Our findings show that transcript levels of endometrial *CAT*, *SOD1* and *SOD2* are similar between pregnant LACT cows and the control pregnancy group represented by inseminated heifers. Interestingly, immediate post calving drying-off leads to lower *CAT* and *SOD1* mRNA levels in endometrial ICAR areas of pregnant DRY cows when compared with pregnant LACT cows. This reduced expression of genes encoding antioxidant enzymes suggests a reduction of ROS and oxidative stress in the endometrium of pregnant cows belonging to the DRY group. Our results provide a solid argument that variations in plasma oxidant status of dry or lactating cows during post-partum take also place in the endometrium. Small perturbations of oxidative stress balance through early fetal life can have profound implications for offspring metabolism and cause diseases in adulthood [31,59]. To understand how modifications in endometrial expression of oxidative stress-related genes impact on conceptus development requires longitudinal studies with comprehensive analyses of the redox environment including markers and enzyme activities at the conceptus-uterus interface when pregnancy establishes.

The forkhead transcription factor FOXL2 has been established as a master gene for folliculogenesis and maintenance of ovarian function in vertebrates [45,60]. Our previous data have shown that *FOXL2* transcript and protein are expressed in bovine and ovine endometrium and are regulated by progesterone but not by oestradiol [28,61]. The current study demonstrated a significant increase in *FOXL2* transcript and protein expression in endometrial ICAR areas of pregnant dry cows compared with those collected from pregnant lactating cows. Therefore regulation of *FOXL2* gene expression appears to be specific to each endometrial area. Compared with our previous report [28], up-regulated expression of *FOXL2* was not a consequence of a drop in progesterone circulating concentrations which were similar in the three experimental groups. Identifying molecular regulators of *FOXL2* production according to the endometrial cell type in the context of metabolism variations will require complementary experiments.

After the pioneering report of Batista *et al*. [34] establishing FOXL2-stimulated *SOD2* expression in a granulosa tumor cell line (KGN cells), subsequent findings have demonstrated that *FOXL2* upregulation promotes granulosa cell accumulation in G1 phase and protects these cells from oxidative damage, notably by stimulating the production of antioxidant molecules [47,62]. Based on these results, we hypothesize a direct link between *FOXL2* and oxidative-related factors in the endometrium of postpartum dairy cows displaying distinct metabolic status. In our bovine model, reduced levels of oxidative stress-related gene transcripts did not correlate with a decrease in *FOXL2* gene expression. Under our experimental conditions, *FOXL2* overexpression did not impact *SOD2*, *SOD1* or *CAT* transcript levels in primary cultures of endometrial fibroblasts or glandular cells. Although interactions between FOXL2 and oxidative stress regulation cannot be ruled out in the endometrium, this connection is not obvious. A comprehensive approach will be necessary to bring new insights to the biological roles of FOXL2 in the endometrium.

Metabolic disorders have been shown to exert detrimental effects on reproduction as illustrated by impaired uterine receptivity and miscarriage in women [63]. Until recently, dairy cattle have been intensively selected to increase milk production with a significant decline in reproductive performances [64,65] that has been extensively investigated at the level of oocyte and embryo quality [66]. Nevertheless recent published data have shown an increased interest for determining the impact of selection on endometrial physiology of high producing dairy cattle [52,67,68]. These reports have focused on the analysis of endometrial ICAR area, whereas our present data have revealed that expression profiles of a selection of genes are distinct between the endometrial CAR and ICAR areas in the post-partum cow associated with metabolic status. In addition, principal component analyses of gene expression in ICAR areas have also revealed that the group of pregnant DRY cows was distinct from the groups of pregnant LACT cows and HEIF. Consequently, the impact of variations in maternal metabolism appears to be more profound on endometrial areas that contain endometrial glands. Endometrial glands are the main producers of histotroph, critical for conceptus elongation in cattle as well as for successful implantation and progression of pregnancy in mammals [69]. Endometrial cell-specific gene expression and histotroph composition will deserve further studies to shed light on the impact of metabolism on uterine secretions relatively to conceptus development.

## Conclusion

The present study has demonstrated that maternal metabolism rather than lactation perturbs endometrial function in cattle. ICAR areas have been identified as the endometrial regions more affected by perturbations in maternal metabolism in post-partum cows. We identified *FOXL2* and oxidative stress-related enzymes as genes whose expression was modified by the absence of lactation. Based on our gene expression data in endometrial ICAR areas of these pregnant females, lactating cows were not significantly different from heifers whereas previously published analyses of circulating metabolites (insulin, IGF1, glucose, NEFA) in these same females indicated that lactating cows were distinct from heifers and dry cows during postpartum in the absence of pregnancy [39]. Pregnancy may have brought about modifications in expressed factors and biological functions in the endometrium of lactating cows that were not detected with our candidate gene approach. Certainly more genes and other biological functions in the endometrium are affected, and their identification prompts the need for more comprehensive molecular analyses of this tissue. Considering the driving property of the endometrium on the trajectory of embryo development [13], it appears critical to determine if molecular modifications detected at the endometrial level have an impact on conceptus features that have been shown to react to subtle modifications in dam energy metabolism [5]. These investigations may provide new approaches for nutritional strategies that could alleviate the negative impact of lactation on prenatal programming of adult performances when lactation and pregnancy overlap.

## Supporting information

S1 Table Primer information used for quantitative real time PCR analysis of bovine candidate genes. All primers were used at a concentration of 300 nM in a final reaction volume of 15 μl.
(XLSX)
